# Supersensitive Detector of Hydrosphere Pressure Variations

**DOI:** 10.3390/s20236998

**Published:** 2020-12-07

**Authors:** Grigory Dolgikh, Sergey Budrin, Stanislav Dolgikh, Aleksandr Plotnikov

**Affiliations:** V.I. Il’ichev Pacific Oceanological Institute FEB RAS, 690041 Vladivostok, Russia; ss_budrin@mail.ru (S.B.); sdolgikh@poi.dvo.ru (S.D.); lotos@poi.dvo.ru (A.P.)

**Keywords:** supersensitive detector, Michelson interferometer, frequency-stabilized helium-neon laser, wind wave, infra-gravity waves, laser strainmeter, low-frequency hydroacoustic emitter

## Abstract

This paper presents an instrument based on an equal-arm Michelson interferometer and a frequency-stabilized helium-neon laser. It is designed to record hydrosphere pressure variations in the frequency range from 0 (conventionally) to 1000 Hz, with accuracy of 0.24 mPa at sea depths of up to 50 m. The operating range of the instrument can be increased by order of magnitude by improving the registration system speed, and accuracy can be enhanced by using larger diameter membranes and/or their smaller thickness. The paper demonstrates some experimental results obtained on the supersensitive detector of hydrosphere pressure variations, confirming its high performance in the infrasonic and sonic ranges.

## 1. Introduction

A human perceives the laws of nature through studying the reasons for changes in its various conditions and this perception is associated, first of all, with the process of observation, study, and analysis of the obtained observation results. It is an enormously laborious and long-term process, the results of which can be expedited through theoretical and model studies, but, nevertheless, the primary role in the world perception belongs to experimental results, the level of which depends on the equipment used in the study.

Studying the processes developing in the World Ocean, we use various equipment to record the spatio-temporal variations of different-scale fields of the World Ocean parameters, of periodic, quasiperiodic, and aperiodic nature in the infrasonic, sonic, and ultrasonic ranges. Whereas the infrasonic range is associated mainly with the processes of inanimate nature, the sonic and ultrasonic ones are associated with both artificial and living objects, emitting communication and location signals. At present, various hydrophone systems and complexes, the recognized world manufacturer of which is Bruel & Kjaer [1], are used to study the processes and the sources of signals in sonic and ultrasonic ranges. To study the nature of variations of the periodic, aperiodic, and quasiperiodic character in the infrasonic range, various wave meters and level meters are used, manufactured by different firms, for example, [2,3,4]. The mareographs, along with wave meters and level meters, are widely used to study oscillations and waves of various ranges. The obtained data, jointly with GPS data, allow sea level changes to be estimated more accurately [5]. The use of various mathematical models together with the data from various level meters makes it possible to find new regularities in the wave processes of the World Ocean [6,7]. The main interest in studies of various processes of the World Ocean is associated with establishing the laws of their occurrence, development, and transformation, with the obligatory determination of the primary source. This task is extremely complicated, since the primary source of the processes observed in the World Ocean can be located in any of the geospheres. For example, let us consider the oscillations and waves of the “Infragravitational noise of the Earth” range (periods of oscillations from 30 s to 10–12 min). There are still various points of view on the nature of oscillations and waves of a given period’s range recorded in the sea. Each of them finds more or less convincing confirmation in the observed experimental data [8]. For example, in Nishida et al. [9], Kobayashi et al. [10], Nishida et al. [11], Tanimoto et al. [12], and Fukao et al. [13], it is stated that variations in atmospheric pressure lead to initiation of the corresponding elastic oscillations of the Earth’s crust, plus, moreover, to variations in hydrosphere pressure at the corresponding frequencies. On the other hand, the variations in atmospheric pressure lead to the initiation of trains of internal sea waves [14]. In addition, the appearance of the “Infra-gravitational noise of the Earth” is associated with sea wave processes: (1) infra-gravitational sea waves [15,16,17,18]; (2) transformation of the energy of internal sea waves into the energy of microdeformations of the Earth’s crust of the corresponding period. A special role in the appearance of oscillations and waves of this range of periods is assigned to processes occurring in the solid spheres of the Earth. Appearance of waves with periods of 100–200 s can be associated with the processes of preparation and development of earthquakes. We know that the bulk of the torsional and spheroidal free oscillations of the Earth are in this range (1–15 min) [19]. 

In studying the regularities of occurrence of oscillations and waves of the World Ocean of the infrasonic and sonic ranges, the technical characteristics of the equipment used in studies are of paramount importance. It should have both the highest sensitivity and the best frequency and dynamic ranges. At present, based on the use of modern laser-interference methods, an instrument has been created for measuring the hydrosphere pressure variations in a wide frequency and dynamic range [20]. Unlike the devices created earlier, this device is based on the equal-arm Michelson interferometer, which made it possible to increase its accuracy by several orders of magnitude. The use of a mechanical temperature compensation system in this device makes it possible to exclude the influence of temperature variations on the device’s readings, thereby increasing the measurement accuracy. Further use of this instrument allowed us to study its capabilities and evaluate its frequency and dynamic ranges. In this paper, its characteristics are considered in detail with estimation of its measuring accuracy. Certain results of measurements in various frequency ranges are presented, demonstrating some of its unique capabilities. 

## 2. Instrument Description. Measuring Accuracy and Errors

**Figure 1:**
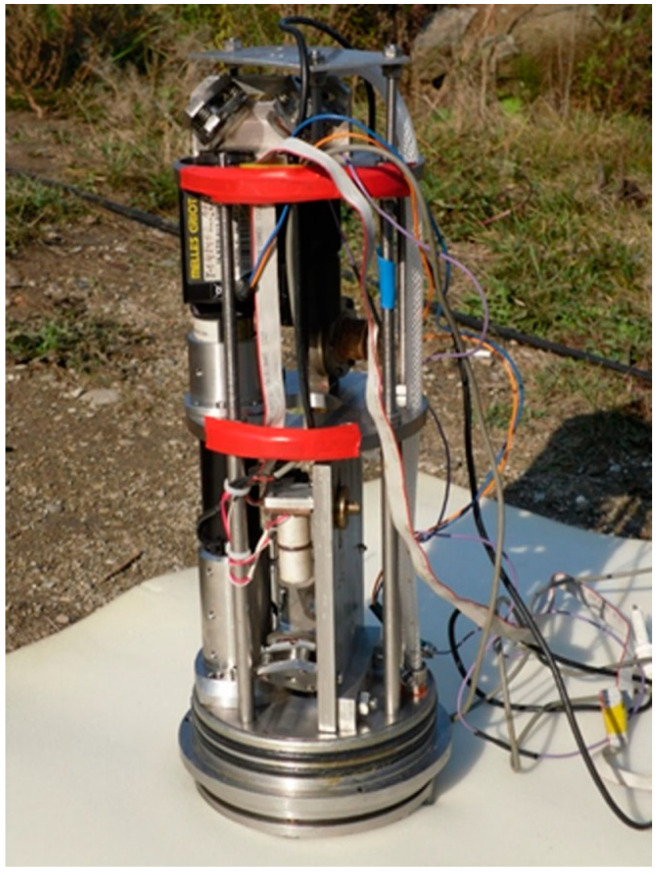
Internal view of supersensitive detector of hydrosphere pressure variations.

Figure 1 shows the internal view of the instrument, created based on the use of a modified Michelson interferometer of the homodyne type and a frequency-stabilized helium-neon laser, which ensures stability of the radiation frequency in the ninth decimal place. It is enclosed in a cylindrical stainless-steel housing, which is fastened in a protective grating designed to protect the instrument in severe operating conditions (rocky or slimy bottom). One side has a hole for cable entry. The other side is sealed with a lid. In addition to the protective grating, an elastic air-filled container is located outside the instrument. Its outlet is connected with a tube to a compensation chamber located in the removable cover. The housing contains a Michelson interferometer, the compensation chamber, an electromagnetic valve, and a digital recording system. 

**Figure 2:**
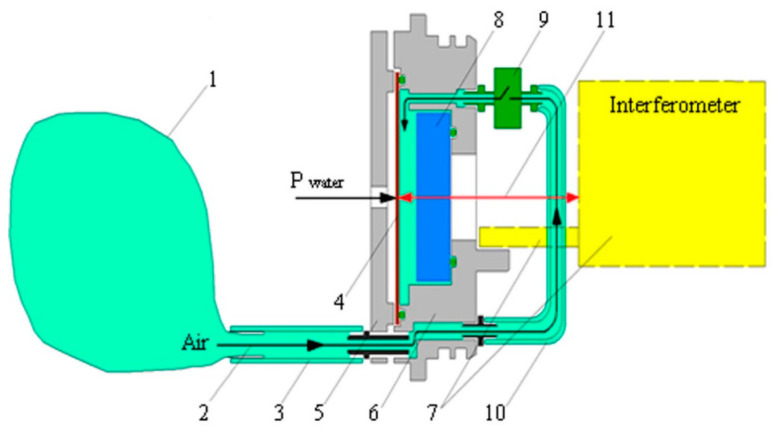
Scheme of external pressure compensation system. 1—air-filled container, 2—direction of air movement, 3—connecting tube, 4—membrane, 5—membrane protection, 6—removable cover base, 7—interferometer, 8—optical window, 9—valve, 10—connecting tube, 11—laser beam.

The sensitive element of the supersensitive detector is the round stainless-steel membrane, which is fixed at the end face of the device. On the outside, the membrane interacts with water. A mirror is fixed on the thin pin inner side of the membrane (Figure 2), which is a part of the “cat’s eye” system, consisting of a biconvex lens with the appropriate focal length and this mirror. The mirror with the lens is included in the structure of the measuring arm of the interferometer. The mirror, rigidly fixed on a thin pin in the center of the membrane, shifts along the interferometer axis under the influence of the hydrosphere pressure variations. A change in the length of the measuring arm leads to the change in the intensity of the interference pattern, recorded by the digital registration system. The output signal of the supersensitive detector, after preprocessing by the digital registration system, is the hydrosphere pressure variations.

An external pressure compensation in-built system is integrated into the instrument (Figure 2) to keep the sensitive element (membrane) in a neutral position when the instrument is being immersed to the operational depth. To keep the membrane (4) in the neutral position during immersion, the electromagnetic valve (9) opens. Air from the outer elastic container (1) under the hydrosphere pressure impact comes into the compensation chamber (8) via the armored tubes (3 and 10). Balancing the external pressure and the pressure inside the chamber brings the membrane to the neutral position. When the instrument is set at the operational depth, the valve closes, and the instrument begins to register the hydrosphere pressure variations. This instrument installation scheme is used for any operational depth, and the membrane is always initially set to the neutral position.

**Figure 3:**
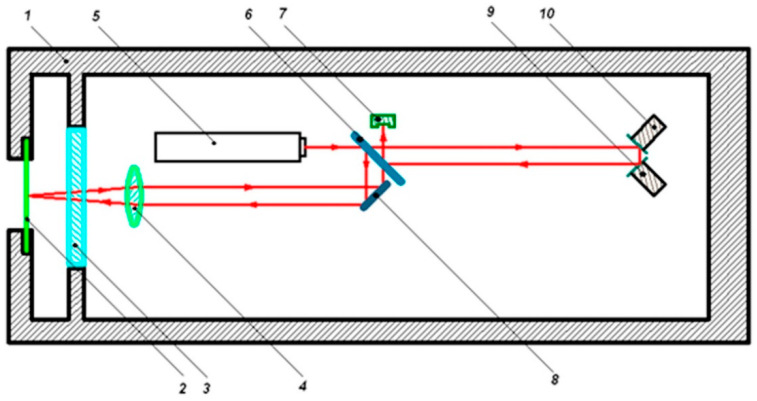
Optical scheme. 1—housing, 2—membrane with a mirror fastened to its the center, 3—optical window, 4—lens, 5—laser, 6—plane-parallel plate dividing plate, 7—photodetector, 8—adjustment reflective mirror, 9—buildup piezoceramics with reflective mirror, 10—compensation piezoceramics with reflective mirror.

Figure 3 shows a simplified optical scheme of the Michelson interferometer, which is implemented in the supersensitive detector. The interferometer uses a frequency-stabilized helium-neon laser by Melles Griott, ensuring the laser radiation frequency stability in the ninth decimal place. The principle of the operation of the interferometer is as follows. The stabilized beam emerges from the laser (5) with a collimator attached to it, and then it is divided into two equivalent parts on the plane-parallel plate (6). The first (reference) part of the beam passes through the build-up (9) and compensation (10) mirrors, mounted on piezoceramic cylinders, and comes to the photodetector (7). The second (measuring) part of the beam by means of an auxiliary mirror (8) passes through the collecting lens (4) and the mirror, rigidly fixed in the center of the membrane, and comes to the photodetector (7). The reference and measuring beams, interacting with each other, form an interference pattern on the photodetector. This Michelson interferometer is a conventional Michelson interferometer of the homodyne type, in which, instead of a triple-prism (reflecting mirror, etc.), a “cat’s eye” system with a mirror fixed on the membrane is used. The interferometer uses the classical method of signal processing, based on phase detection, which currently allows the change in the difference of interferometer arms’ length to be measured with an accuracy of 1 pm. However, this accuracy may be lower due to the presence of external noise, the level of which may be higher. In this installation, an alternating voltage with frequency of 100 kHz is applied to the swinging piezoceramic (9). If necessary, this allows the operating frequency range to be significantly expanded to 10 kHz instead of 1 kHz. This may be necessary when solving various hydroacoustic problems, as well as problems related to the study of communication signals of marine mammals, and also signals generated by other marine biological objects. Laser beams, passing through the reference arm (6, 9, 10) and the measuring arm (6, 8, 4, 3, 2), collide on the photodetector (7), forming there an interference pattern modulated by the swing voltage. This modulation is necessary to determine the value of phase change between the reference and measurement beams and to determine the direction of this change, which is performed by the digital recording system. All structural blocks of the digital recording system (reference signal generator, phase detector, level reset system, etc.) are assembled on microprocessor bases and 24-bit digital-to-analog converters. The use of modern high-speed microprocessors and 24-bit digital-to-analog converters has significantly expanded the operating range of the sensor and increased its sensitivity. The operating dynamic range of the interferometer extends from 0.315 μm (the distance from one maximum of the interference pattern to any neighboring one) to the smallest quantum value determined by the speed of microprocessors and 24-bit digital-to-analog converters. Taking into account the need to assign two digits under the signs (+ or −), the smallest quantum value will be 0.075 pm. This value is still unattainable, which is associated with a high noise level of photoelectronic equipment, laser frequency instability, variations in laser radiation power, etc. The reduction of this noise due to the use of the Michelson equal-arm interferometer scheme allows us to hope that the limiting value of the laser beam phase incursion measurement will be achieved.

At the moment of the first activation of the supersensitive detector at the operational depth, the interference pattern is adjusted to the maximum intensity. The hydrosphere pressure variations shift the membrane center relative to the neutral position to one or the other side, thereby decreasing or increasing the measuring arm length. These variations change the intensity of the interference pattern on the photodetector. The digital registration system controls the interferometer, maintaining the maximum intensity of the interference pattern. The photodetector records the change in the interference pattern brightness and transmits the signal to the digital registration system, which puts high voltage out to the piezoceramic compensation cylinder. Under the action of the voltage applied to the piezoceramic cylinder, its geometric dimensions change, which leads to increase or decrease in the length of the reference arm. The change in the measuring arm length is compensated by the reference arm length, maintaining the interference pattern intensity at the maximum. The output interferometer signal is the voltage applied to the piezoceramic compensation cylinder, directly proportional to the change in the measuring arm length. When the change in the length of the interferometer measuring arm becomes equal to 0.315 μm (half the wavelength of the helium-neon laser), the voltage on the compensation piezoceramics is dropped in the registration system. In this case, the system jumps from the operation maximum of the interference pattern intensity to one of the two neighboring ones. By the direction of the membrane center displacement, the registration system determines to which maximum of the two (left or right). During the operation of the supersensitive detector, there can be a lot of such drops. The recorded signal consists of the voltage of the sum of these drops and the voltage after the last one. Thus, the large dynamic range of the supersensitive detector, limited only by the membrane mechanical properties, is realized.

The measuring accuracy of the membrane displacement is limited, on one hand, by the frequency stability of the laser used, which can be calculated by the formula

(1) $$\Delta l=L\frac{\Delta \nu }{\nu },$$

where L is the difference in optical path in the interferometer arms at the initial moment of measuring, and $\frac{\Delta \nu }{\nu }$ is frequency stability. With the stability of the laser radiation frequency in the ninth decimal place, and with different lengths of the reference and measuring arms equalizing up to 1 sm, we have measuring accuracy of the membrane center displacement above the noise, caused by the instability of the laser radiation frequency, which equals Δ*l* = 10^−11^ m. In this Michelson interferometer, in addition to the noise caused by the instability of the laser radiation frequency, there are noises caused by the photoelectronic equipment, Δ*i*_1_, and the stability of the laser radiation power, Δ*i*_2_. The error caused by the instability of the laser radiation power can be written as $\frac{\Delta i_{2}}{i_{0}}\times \frac{\lambda }{2\pi }$. For a helium-neon laser, it is as follows:$\frac{\Delta i_{2}}{i_{0}}\approx 0.0001$ and then, $\frac{\Delta i_{2}}{i_{0}}\times \frac{\lambda }{2\pi }\approx 0.01$ nm. The error caused by the noise of the photoelectronic equipment can be estimated by the formula

(2) $$\Delta l_{1}=\frac{1}{4\pi }\times \sqrt{\left(\frac{\lambda \times h\prime \times c\times \Delta f}{q\times P\prime_{0}}\right)},$$

where: *λ*—wave length, *h’*—Planck’s constant, *c*—speed of light, Δ*f—*band width of the received frequencies, *q*—photodetector quantum efficiency, *P’*_0_—power of laser radiation. Assuming *P’*_0_ = 0.001 W, *q* = 0.25, *c* = 3 × 10^8^ m/s, *λ* = 0.63 × 10^−6^ m, *h’* = 6.626 × 10^−34^ J × s, we have

(3) $$\Delta l_{\min}=1.78\times 10^{-15}\frac{1}{4\pi }\times \sqrt{\Delta f}\;m/{\mathrm{Hz}}^{1/2}$$

For the helium-neon laser at Δ*f* = 104 Hz, we have Δ*l_min_* = 1.78 × 10^−13^ m.

Thus, the inaccuracy caused by the error in determination Δ*l* does not exceed the measurement accuracy of the change in the difference of the interferometer arms’ lengths [21].

Next, let us determine the minimum change in hydrosphere pressure that can be detected by the supersensitive detector with the above-mentioned stability of the operating laser used. To do this, let us use the formula for the circular membrane fastened at the edges [22]:

(4) $$\Delta P=\frac{16\times \Delta l\times h^{3}\times E}{3\times (1-\sigma^{2})\times R^{4}},$$

where Δ*l* is membrane center displacement, *h* is membrane thickness, *E* is the Young’s modulus, *σ* is the Poisson ratio, and *R* is membrane diameter.

The instrument can use the membranes of different thickness, made of stainless steel. For *R* = 50 mm, *h* = 0.5 mm, *E* = 2.1 × 10^11^ N/m^2^, *σ* = 0.25, we have

Δ*P =* 2.4 × 10^7^ Δ*l* Pa (5)

Further, based on Equation (1) and the condition that the difference in the optical path in the interferometer arms does not exceed 1 cm, we get

Δ*P* = 2.4 × 10^−4^ Pa (6)

By equalizing the arm lengths of the supersensitive detector of hydrosphere pressure variations with greater accuracy and using membranes of smaller thickness (or larger diameter), its sensitivity can be significantly improved. The measured maximum change in hydrosphere pressure is limited, first of all, by mechanical strength characteristics of the membrane.

To determine the measurement error, we use the expressions given in [21]. The calculations are carried out under the condition that the membrane is made of stainless steel with the thermal expansion coefficient α = 1.1 × 10^−5^ m/°C. We get that the error of the instrument associated with change in the membrane thickness is equal to 7.9 × 10^−10^ Pa, when the temperature changes by 0.1 °C, and the error associated with change in the membrane radius, when the temperature changes by 0.1 °C, is equal to 1.1 × 10^−9^ Pa. Under the condition that all parts of the interferometer are made of invar with a coefficient of thermal expansion α = 5 × 10^−7^ m/°C, and when equalizing the lengths of the measuring and reference arms to 10^−4^ m, the measurement error, when the temperature changes by 0.1 °C, is by 3 orders of magnitude smaller. In our calculations, we took a temperature change of 0.1 °C, which is quite justified. Measurements are taken at sea depths greater than 10 m, where temperature variations are very small. Large variations in temperature can only be caused by infrequent seasonal internal waves. In addition, changes in external temperature can practically only affect the membrane. Other structural elements of the interferometer are located inside a cylindrical body, the walls of which are more than 1 cm thick. Under these conditions, rapid temperature changes practically do not affect the internal elements of the interferometer. Typically, these temperature changes can be caused by quasi-periodic internal waves with periods ranging from 4 to 15 min.

**Figure 4:**
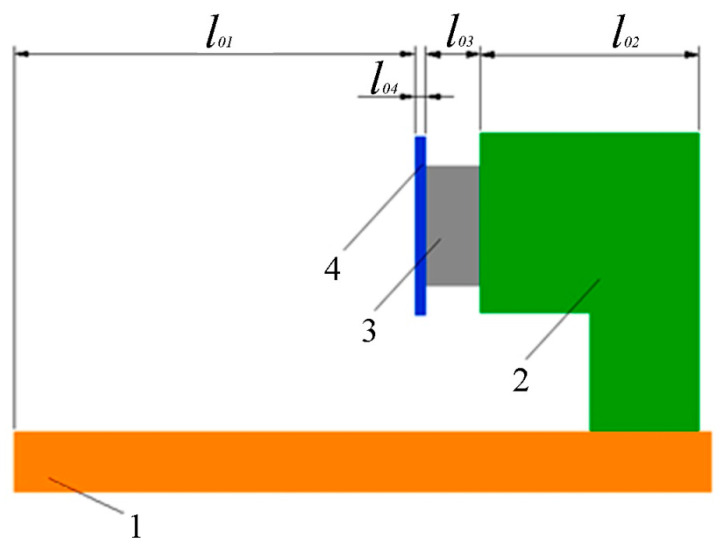
Example of optical bench section. 1—bench (*α*_1_), 2—fasteners (*α*_2_), 3—piezoceramics (*α*_3_), 4—mirror (*α*_4_).

Using materials with different thermal expansion coefficients and making optical parts of special shapes, we can compensate for changes in the interferometer geometric dimensions. Figure 4 shows a diagram of mounting the mirrors on the piezoceramic cylinders.

The length of the mounting unit (*l*_02_) is selected so as to compensate for the change in the optical bench section length (Δ*l*_01_), taking into account the change in the mirror thickness (Δ*l*_04_) and the piezoceramic cylinder length (Δ*l*_03_). As a result, we get

(7) $$l_{02}=\frac{l_{01}\times \alpha_{1}-l_{03}\times \alpha_{3}-l_{04}\times \alpha_{4}}{\alpha_{2}}.$$

Knowing the lengths of all components and taking into account the thermal expansion coefficients of each part, we can select the length of the part *l*_02_ in such a way as to exclude any temperature effect on the change in the optical path of the beams.

Knowing the lengths of all of the interferometer parts and the angles of their rotation relative to the laser beam, we can choose the materials for the parts so as to compensate for any temperature change impact on the length of the interferometer reference and measuring arms.

## 3. Demonstration Results of Supersensitive Detector Use

**Figure 5:**
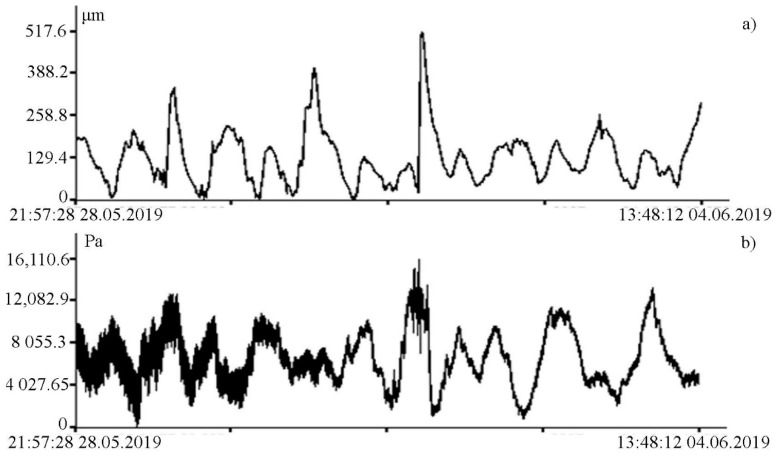
Synchronous records of the 52.5 m laser strainmeter (**a**) and the supersensitive detector of hydrosphere pressure variations (**b**).

**Figure 6:**
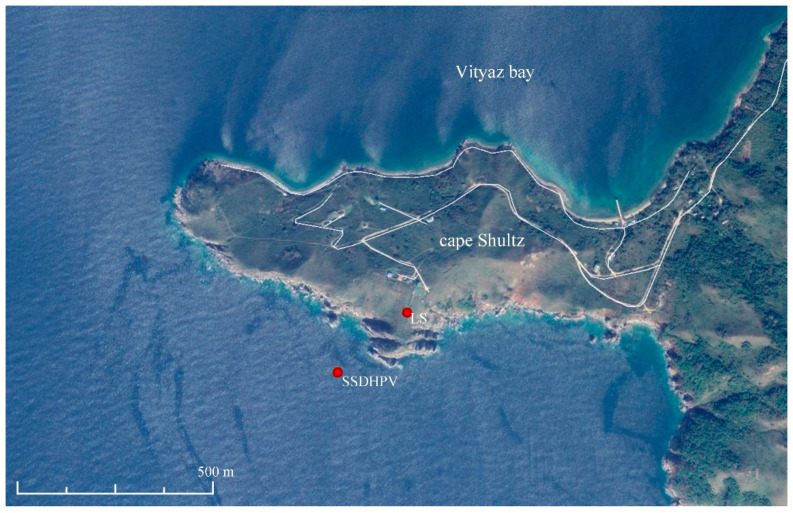
Schematic map of location of the supersensitive detector of hydrosphere pressure variations (SSDHPV) and the 52.5 m laser strainmeter (LS).

Figure 5 shows the synchronous record fragments of the supersensitive detector of hydrosphere pressure variations moored on the shelf of the Sea of Japan at a depth of 27 m and with a 52.5 m laser strainmeter installed in an underground room at Schultz Cape (see Figure 6).

**Figure 7:**
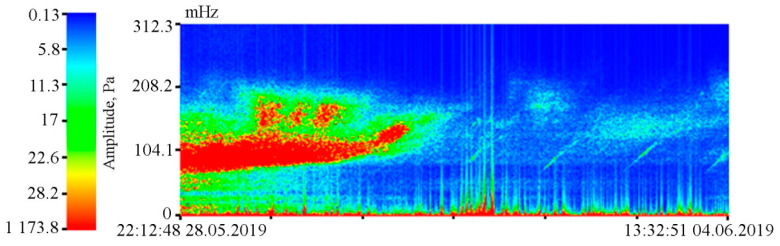
Dynamic spectrogram of the supersensitive detector of hydrosphere pressure variations and a legend.

As we can see in Figure 5, both instruments reliably record ultra-low-frequency oscillations caused by the tidal effect. During spectral processing of the detector record, it is established that the amplitude of the 12 h tide is higher than that of the 24 h tide, which is typical for the irregular tide of the Sea of Japan [23]. However, during spectral processing of the laser strainmeter record, the amplitude ratios of the 24 and 12 h tides are opposite. This is due to the fact that the amplitudes of 24 and 12 h tides in the Earth’s crust recorded by the laser strainmeter are opposite to the amplitudes of the tides of the Sea of Japan. The supersensitive detector record shows a significant broadening of line, caused by sea waves associated with incoming and local wind waves. The dynamic spectrogram (see Figure 7) clearly shows a change in the period of wind waves, recorded by the detector, associated with both dispersion and other linear and nonlinear processes. Figure 7 shows the registration of swell waves, in the initial stage with a period of about 12 s, the period of which gradually decreased to 6 s. In the dynamic spectrogram, there is occasional registration of local wind waves with periods of 3–5 s.

**Figure 8:**
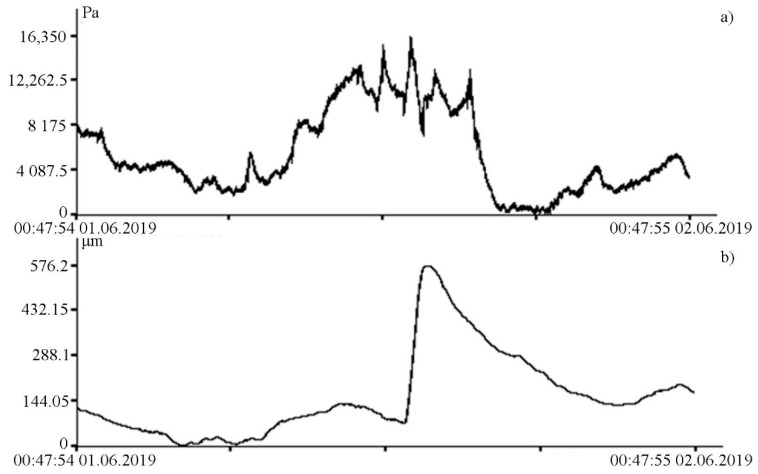
Synchronous record fragments of the supersensitive detector of hydrosphere pressure variations (**a**) and the 52.5 m laser strainmeter (**b**).

It is especially interesting to use the supersensitive detector of hydrosphere pressure variations in combination with other devices that measure changes in various parameters in neighboring geospheres. Such complex experiments allow the causal relationships of various geospheric processes and phenomena of wide frequency ranges to be investigated and their primary source to be determined. As a typical example of the profitable conduct of such experiments, we can cite some results, obtained during synchronous experiments on the detector of hydrosphere pressure variations and the 52.5 m laser strainmeter. Thus, in the infrasonic range, interesting disturbances of hydrosphere pressure were noted, characteristic for a group of solitary waves (see Figure 8a), which caused disturbance of the Earth’s crust in a soliton-like shape (see Figure 8b).

**Figure 9:**
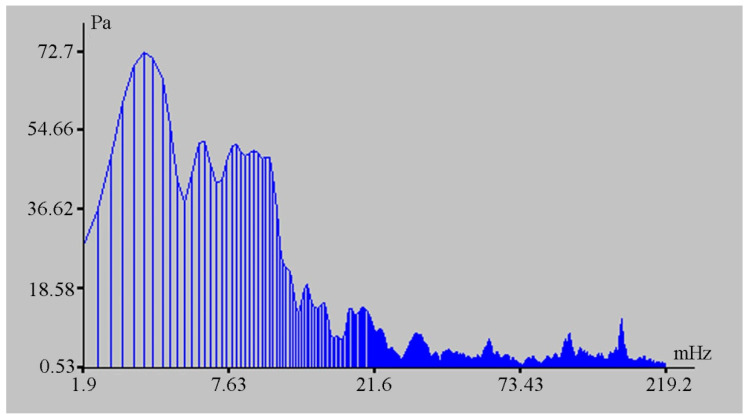
Spectrum of the detector of hydrosphere pressure variations record fragment.

**Figure 10:**
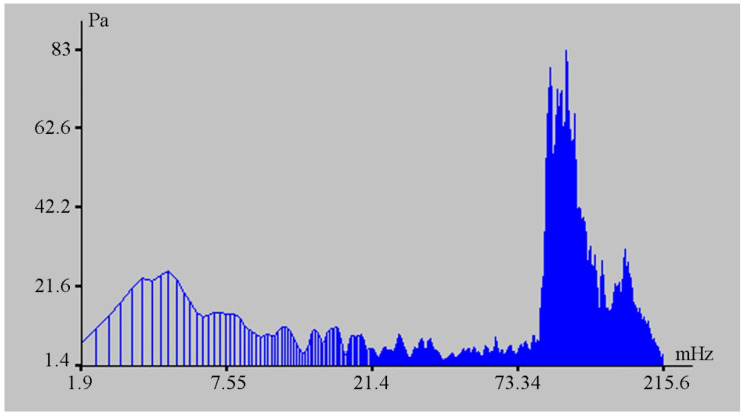
Spectrum of the detector of hydrosphere pressure variations record fragment.

The change in the hydrosphere pressure was about 14 kPa, which caused a change in the displacement on the laser strainmeter bases of the order of 500 μm. The magnitudes of these disturbances are greater than the tidal effect. From the laser strainmeter and the detector of hydrosphere pressure variations data, we can estimate the loading effect of marine processes on the deformation of the upper layer of the Earth’s crust. In this case, it is equal to 35.7 nm/Pa. During this observation period, powerful spectral components in the infra-gravitational range of periods (2–6 min) are singled out on the spectra of the detector of hydrosphere pressure variations record fragments, the amplitudes of which are significantly higher than the amplitudes of wind sea waves (6–11 s) (see Figure 9), in contrast to other records of the detector of hydrosphere pressure variations, when the amplitudes of wind waves are significantly higher than the amplitudes of waves in the infra-gravitational range (see Figure 10).

**Figure 11:**
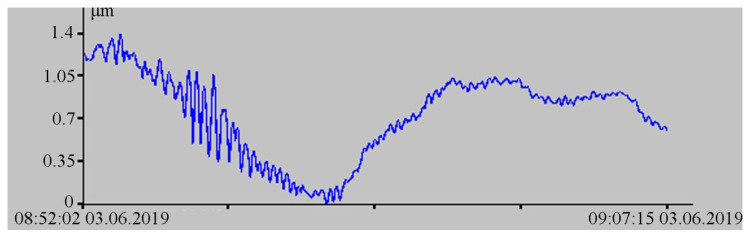
Fragment of the 52.5 m laser strainmeter record.

The importance of an integrated approach to the interpretation of the obtained experimental data can be demonstrated with the example of interpretation of the obtained experimental data of the 52.5 m laser strainmeter. In Figure 11 of the laser strainmeter record fragment, there is a signal, the appearance of which is specific for a small earthquake that occurred at some distance from the registration area.

**Figure 12:**
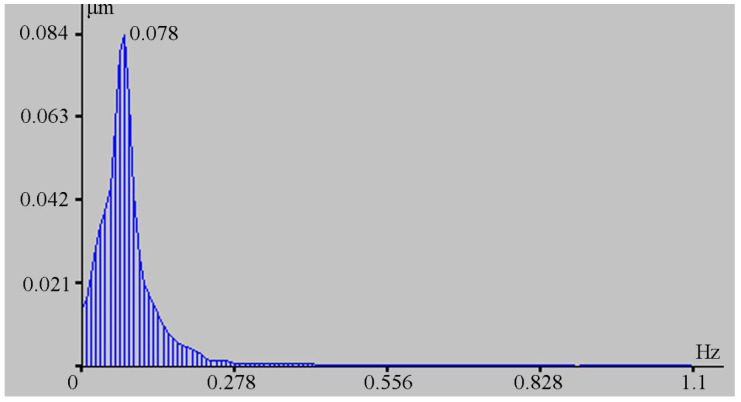
Spectrum of the fragment of the 52.5 m laser strainmeter record.

During spectral processing of successive individual fragments of this record, it is established that the main energy maximum varies within small ranges from 12.8 to 10.5 s. Since this range of periods is also typical for marine infragravity waves that contribute to the instrument records, there is no clear dispersion dependence in the behavior of the selected maxima over time. Figure 12 shows a typical spectrum of the laser strainmeter record fragment, obtained from 256 points with a sampling rate of 2.2 Hz, on which a peak with a period of 12.8 s (0.078 Hz) stands out.

**Figure 13:**
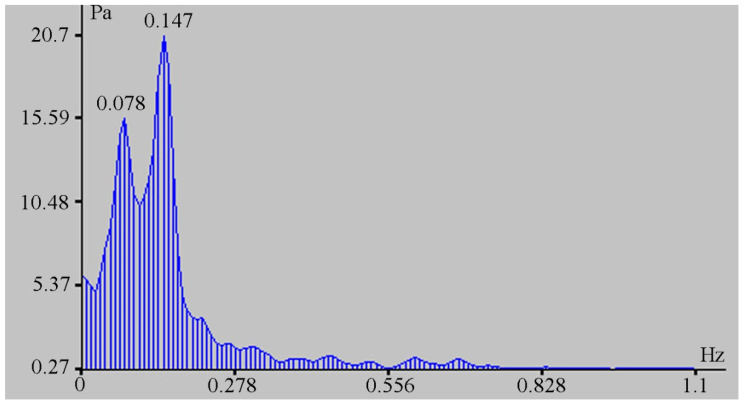
Spectrum of the record fragment of the supersensitive detector of hydrosphere pressure variations.

The processed record fragment began at 08 h 53 min 35 s and ended at 08 h 55 min 30 s on 3 June 2019. During processing of the synchronous record of the supersensitive detector of hydrosphere pressure variations, the presence of oscillations with characteristic periods, singled out from the laser strainmeter record, was established, but time-wise they were recorded much later than the oscillations singled out on the laser strainmeter. Figure 13 shows the spectrum of the initial fragment of the detector of hydrosphere pressure variations record, which began at 09 h 32 min 10 s and ended at 09 h 34 min 06 s, where there is a characteristic peak with a period of 12.8 s (0.078 Hz).

To the right of this peak, there is a peak with a period of 6.8 s (0.147 Hz), caused by surface wind waves of this zone of the Sea of Japan. The interval in recording these oscillations by the laser strainmeter and the detector of hydrosphere pressure variations is about 38 min 35 s. Detection of synchronous oscillations with such a delay indicates that the source of oscillations is in water and the signal has come through the Earth’s crust to the laser strainmeter and through the water to the detector of hydrosphere pressure variations. In accordance with Dolgikh et al. [24], we calculate the speed of a surface wave in deep water using the formula

(8) $$c=\frac{gT}{2\pi },$$

where *g* = 9.8 m/s^2^, *T* is the period, and *π* = 3.14. At the wave period of 12.8 s, the wave speed is 20 m/s. If we take the speed of the Rayleigh wave in the surface layer of the Earth’s crust as equal to 2000 m/s, then we can calculate the location of the source of oscillations with a period of 12.8 s, which is about 46.8 km. From the comparison of the obtained results, we can affirm that an earthquake cannot be the source of the signal recorded by the laser strainmeter.

**Figure 14:**
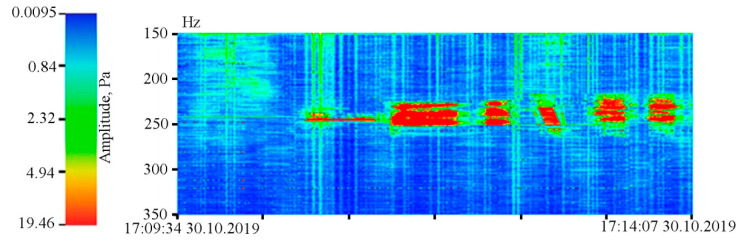
Dynamic spectrogram of the record of the supersensitive detector of hydrosphere pressure variations, obtained in the process of recording signals of various complexity, generated by a low-frequency hydroacoustic emitter with carrier frequency of 245 Hz.

In conclusion, let us give examples of recordings by the supersensitive detector of hydrosphere pressure variations of the hydroacoustic oscillations generated in water by various low-frequency hydroacoustic emitters. Figure 14 shows the dynamic spectrogram of the detector of hydrosphere pressure variations record, obtained when the hydroacoustic emitter was operating at the frequency of 245 Hz in the process of emitting a standard signal package of various complexity. From left to right: harmonic signal with a duration of 30 s, phase-shift keyed signal (M-sequence) with a duration of 30 s, phase-shift keyed signal (M-sequence) with a duration of 10 s, sweep signal with a duration of 10 s, two phase-shift keyed signal (M-sequence) with a duration of 15 s.

**Figure 15:**
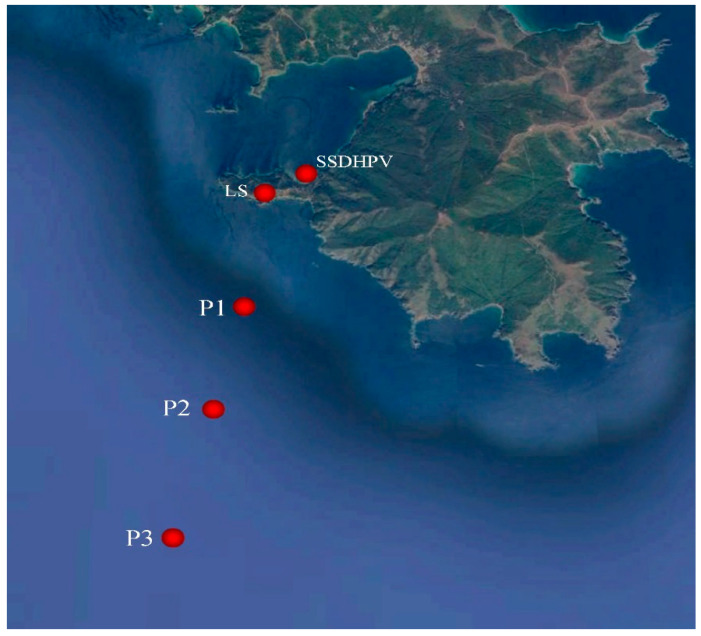
Experiment schematic. SSDHPV—supersensitive detector of hydrosphere pressure variations location. LS—52.5 m laser strainmeter location. P1–P3—emitting stations.

**Figure 16:**
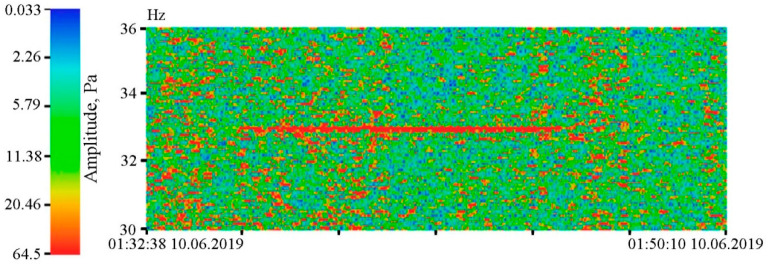
Dynamic spectrogram of the fragment of the supersensitive detector of hydrosphere pressure variations record, when a low-frequency hydroacoustic emitter is operating at the frequency of 33 Hz.

**Figure 17:**
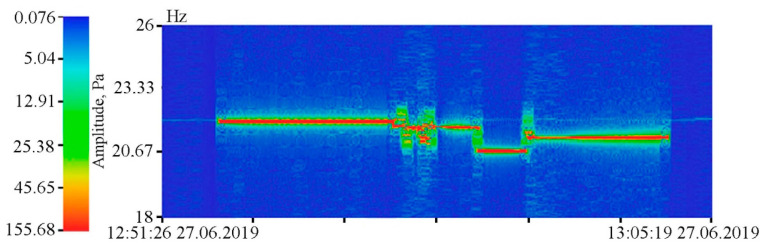
Dynamic spectrogram of the fragment of the supersensitive detector of hydrosphere pressure variations record, when a low-frequency hydroacoustic emitter is operating at the frequency of 22 Hz, with slight variations in the emitting frequency.

In the summer of 2019, we researched the regularities of low-frequency hydroacoustic signals propagation from the open sea to a semi-closed bay through the land part of Shultz Cape. Generation of low-frequency harmonic signals was made using two hydroacoustic emitters of the electromagnetic type, operating at frequencies of 33 [24] and 22 Hz [25]. The detector of hydrosphere pressure variations was located inside the Vityaz Bay. The entire schematic of the experiment is shown in Figure 15, and Figure 16 and Figure 17 show the dynamic spectrograms obtained when processing the records of the detector of hydrosphere pressure variations during the operation of hydroacoustic emitters at the station P3, at frequencies of 33 and 22 Hz, respectively. The power of the signal emitted at a frequency of 33 Hz is almost 3 times less than the power of the signal emitted at a frequency of 22 Hz. Accordingly, the amplitudes of the received signals are different. The change in the frequency of the received signal in Figure 17 is due to the uneven operation of the hydroacoustic emitter.

## 4. Conclusions

The developed and created supersensitive detector of hydrosphere pressure variations based on an equal-arm Michelson interferometer, which has unique amplitude-frequency characteristics, is designed to measure variations of hydrosphere pressure with nanoscale accuracy in the infrasonic and sonic ranges within a large dynamic range. Its main technical characteristics (operating range: 0 (conventionally)–1000 Hz, measuring accuracy of hydrosphere pressure variations—0.24 mPa, operating depths—up to 50 m). It is possible to obtain the limiting technical characteristics with a decrease in the noise of photoelectronic equipment, compensation for temperature noise, more accurate equalization of the difference in the lengths of the measuring and reference arms of the interferometer, which correspond to the following calculated parameters: 0 (conventionally)–10,000 Hz, the accuracy of measuring variations in hydrospheric pressure—1.8 μPa.

This instrument can be used in the studies of fundamental and applied nature, aimed at obtaining unique results in wide frequency and dynamic ranges.
